# Comparison of the effects of losartan potassium and benazepril in the treatment of hypertensive patients with insulin resistance

**DOI:** 10.12669/pjms.40.8.9896

**Published:** 2024-09

**Authors:** Tingting Ma, Yangui Wang, Haixia Liu, Tao Wang, Yue Zhao, Yao Li

**Affiliations:** 1 Tingting Ma Department of General Practice, Shanghai Pudong New Area People’s Hospital, Shanghai, 201299, P.R. China; 2 Yangui Wang Department of General Practice, Shanghai Pudong New Area People’s Hospital, Shanghai, 201299, P.R. China; 3 Haixia Liu Dept. of Geriatric, Shanghai Fourth People’s Hospital affiliated to Tongji University, Shanghai, 200081, P.R. China; 4 Tao Wang Department of General Practice, Shanghai Pudong New Area People’s Hospital, Shanghai, 201299, P.R. China; 5 Yue Zhao Department of Cardiology, Yangpu Hospital, School of Medicine, Tongji University, Shanghai, 200090, P.R. China; 6 Yao Li Department of Cardiovascular Medicine, Shanghai Pudong New Area People’s Hospital, Shanghai, 201299, P.R. China

**Keywords:** Losartan potassium, Benazepril, Hypertensive, Insulin resistance

## Abstract

**Objective:**

To compare the efficacy of losartan potassium (LP) and benazepril in the treatment of hypertensive patients with insulin resistance (IR).

**Methods:**

This is a retrospective analysis of the clinical data of 155 hypertensive patients with IR admitted to Shanghai Pudong New Area People’s Hospital from March 2021 to March 2023. Of these 76 received LP treatment (LP group), and 79 received benazepril treatment (benazepril group). Blood pressure levels, blood glucose and insulin levels, treatment efficacy, and incidence of adverse reactions before and after the treatment in both groups were compared.

**Results:**

After the treatment, diastolic and systolic blood pressure in the two groups significantly decreased compared to pre-treatment levels (*P*<0.05), with no significant difference between the two groups (*P*>0.05). After the treatment, levels of fasting plasma glucose (FPG), 2-hours plasma glucose (2hPG), fasting insulin (FINS), 2-hours insulin (2hINS), and insulin sensitivity index (ISI) in both groups significantly decreased compared to pre-treatment levels (*P*<0.05), with no significant difference between the two groups (*P*>0.05). There was no significant difference in the total efficacy and the incidence of adverse reactions between the two groups (*P*>0.05).

**Conclusions:**

The efficacy of LP and benazepril in treating hypertension with IR is equivalent. Both are safe and can effectively lower blood sugar and insulin levels, alleviate IR, and lower blood pressure.

## INTRODUCTION

Insulin resistance (IR) refers to a series of pathological and physiological changes caused by the loss or decrease of insulin biological efficacy response due to the influence of target tissue factors in the body.^1^ It is manifested by a decrease in the utilization of glucose by body tissues (especially adipose and muscle tissue) and a decrease in liver glucose output.^1^,^2^ Studies have shown that IR is an important pathophysiological basis for the occurrence of complications such as obesity, coagulation disorders, impaired glucose tolerance, abnormal fasting blood glucose, and dyslipidemia in hypertensive patients.^3^,^4^

IR is considered one of the important factors in the onset of hypertension, and the incidence of IR in patients with hypertension can reach 50%.^5^,^6^ Therefore, the current clinical treatment of hypertension has gradually shifted from simple hypotension to metabolic treatment.^7^,^8^ Losartan potassium is an angiotensin II receptor blockers (ARB) and is effective as a once-daily antihypertensive agent.^9^,^10^ Benazepril belongs to the family of angiotensin-converting enzyme inhibitors (ACEi), which lowers blood pressure and increases the supply of blood and oxygen to the heart and can be used either alone or in combination with other antihypertensive medications.^11^

However, there is limited literature comparing the therapeutic effects of these two drugs in hypertensive patients with IR. The aim of this study was to review and analyze the efficacy of LP and benazepril in the treatment of hypertensive patients with IR so as to clarify their clinical application value and to provide practical reference for the clinical treatment of hypertensive patients with IR.

## METHODS

This is a retrospective analysis of medical records of 155 hypertensive patients with IR admitted to Shanghai Pudong New Area People’s Hospital from March 2021 to March 2023. A total of 76 patients received treatment with LP (LP group) and 79 patients received benazepril treatment (benazepril group).

### Ethical Approval:

The ethics committee of Shanghai New Area People’s Hospital approved this study with the number 2023-LW-05 on December 11, 2023.

### Inclusion criteria:

- Patients who were diagnosed with primary hypertension.^12^
- Patients who also met the diagnostic criteria for IR.^13^
- Complete clinical data.

### Exclusion criteria:

- Patients with cardiovascular and cerebrovascular diseases.
- Patients with organic lesions such as kidney and liver.
- Women during lactation and pregnancy.
- Patients with connective tissue lesions.
- Patients with infectious diseases.
- Secondary hypertension patients.
- Patients with other diseases that affect glucose and lipid metabolism.

Losartan Potassium (Zhejiang Huahai Pharmaceutical Co., Ltd.) was administered orally once a day, 50 mg/dose, for one month as a course of treatment, for a total of three courses. Benazepril (Beijing Novartis Pharmaceutical Co., Ltd.) was administered orally once a day, 10 mg/dose, for one month as a course of treatment, for a total of three courses.

### Observation indicators:

- Blood pressure, including diastolic and systolic blood pressure. Patients were instructed to rest for 15 minutes before testing. A mercury column sphygmomanometer was used to measure sitting blood pressure. Measurements were repeated three times with an interval of two minutes each time and the average value was taken.
- Blood glucose and insulin levels, including fasting blood glucose (FPG), fasting plasma glucose (FPG), two hours plasma glucose (2hPG), fasting insulin (FINS), 2-hours insulin (2hINS), and insulin sensitivity index (ISI). FPG and 2hPG were measured using the GOD-PAP method; FINS and 2hINS were measured using radioimmunoassay (kits were purchased from Wuhan Merck Biotechnology Co., Ltd). ISI=In [1/(FPG/FINS)].^14^
- Therapeutic effects. Significant effect: a decrease in systolic blood pressure of ≥ 20 mmHg, a decrease in diastolic blood pressure of ≥ 10 mmHg or return to normal, and a decrease in ISI of ≥ 20%; Effective: reduces systolic blood pressure by 10-19 mmHg, diastolic blood pressure by less than 10 mmHg, and ISI by 10% to 19%; Invalid: does not meet the above standards; Total effective rate of treatment = (significant + effective) / total number of cases × 100%.
- The incidence of adverse reactions.

### Statistical analysis:

All data analysis was conducted using SPSS 22.0 software (IBM Corp, Armonk, NY, USA). The normality of the data was evaluated using the Shapiro Wilk test. The data of normal distribution were represented by mean ± standard deviation, independent sample t-test was used for inter group comparison, and paired t-test was used for intra group comparison before and after the treatment. Non normal distribution data were represented by median and interquartile intervals, and inter group comparisons were conducted using Mann Whitney U test. The counting data were represented by the number of cases and compared using chi square test or continuity correction. *P*<0.05 meant statistically significant difference.

## RESULTS

A total of 155 hypertensive patients (91 males and 64 females) were included in the study. Age of the patients ranged from 39 to 80 years, with a mean age of 59.95 ± 8.88 years. The course of the disease was 1.5-14 years, with a median of 5.4 (4-8) years. There was no significant difference in the general data between the two groups (*P*>0.05) (Table-I). Before the treatment, there was no statistically significant difference in the levels of diastolic and systolic blood pressure between the two groups (*P*>0.05). After the treatment, levels of diastolic and systolic blood pressure in the two groups significantly decreased compared to pre-treatment levels (*P*<0.05), but there was no statistically significant difference between the groups (*P*>0.05) Table-II.

**Table-I:** Comparison of General Information between two groups.

Group	n	Gender (male/female)	Age (years)	Course of Disease (Year)	BMI (kg/m²)
LP group	76	46/30	60.87±8.72	5 (4-7)	24.93±3.35
Benazepril group	79	45/34	59.08±9.00	6 (5-8)	25.84±3.24
*χ*^2^/t/Z		0.203	1.259	-1.863	-1.703
P		0.652	0.210	0.062	0.091

BMI: Body Mass Index.

**Table-II:** Comparison of blood pressure levels between two groups (mmHg).

Time	Group	n	Diastolic pressure	Systolic pressure
Before treatment	LP group	76	108.80±13.11	174.63±14.16
	Benazepril group	79	112.09±14.20	178.30±15.07
	t		-1.496	-1.563
	P		0.137	0.120
After treatment	LP group	76	76.03±8.99^a^	127.34±10.06^a^
	Benazepril group	79	78.16±7.67^a^	129.38±10.36^a^
	t		-1.595	-1.242
	P		0.113	0.216

***Note:*** Compared with the same group before treatment,

a P<0.05.

Before the treatment, levels of FPG, 2hPG, FINS, 2hINS, and ISI between in both groups were comparable (*P*>0.05). After the treatment, FPG, 2hPG, FINS, 2hINS, and ISI in both groups significantly decreased compared to pre-treatment levels (*P*<0.05), with no statistically significant difference between the two groups (*P*>0.05) Table-III.

**Table-III:** Comparison of blood glucose and insulin levels between two groups.

Time	Group	n	FPG (mmol/L)	2hPG (mmol/L)	FINS (mmol/L)	2hINS (mmol/L)	ISI
Before treatment	LP group	76	5.74±0.55	8.37±0.72	21.94±3.58	78.22±9.74	-4.68±0.45
	Benazepril group	79	5.69±0.68	8.42±0.84	21.47±3.65	81.01±11.13	-4.62±0.48
	t		0.501	-0.392	0.811	-1.657	-0.860
	P		0.617	0.695	0.419	0.100	0.391
After treatment	LP group	76	5.11±0.50^a^	7.24±0.70^a^	17.48±3.16^a^	58.39±8.50^a^	-4.09±0.41^a^
	Benazepril group	79	5.07±0.60^a^	7.28±0.80^a^	17.17±3.19^a^	60.19±9.14^a^	-4.01±0.46^a^
	t		0.521	-0.366	0.612	-1.265	-1.146
	P		0.603	0.715	0.541	0.208	0.254

***Note:*** Compared with the same group before treatment,

a P<0.05; FPG: fasting plasma glucose; 2hPG: 2-hours plasma glucose, FINS: fasting insulin, 2hINS: 2-hours insulin; ISI: insulin sensitivity index.

There was no statistically significant difference in the total efficacy of the LP group (93.42%) compared to the benazepril group (91.14%) (*P*>0.05) Table-IV. There was no statistically significant difference in the incidence of adverse reactions between the LP (2.63%) and the benazepril group (5.06%) (*P*>0.05) Table-V.

**Table-IV:** Comparison of treatment effects between two groups.

Group	n	Significant effect	Effective	Invalid	Total effective rate (%)
LP group	76	40 (52.63)	31 (40.79)	5 (6.58)	71 (93.42)
Benazepril group	79	38 (48.10)	34 (43.04)	7 (8.86)	72 (91.14)
χ^2^					0.282
P					0.595

**Table-V:** Comparison of adverse reaction rates between two groups.

Group	n	Dizzy	Headache	Nausea	Vomit	Gastrointestinal reaction	Total occurrence rate (%)
LP group	76	1 (1.32)	0 (0.00)	1 (1.32)	0 (0.00)	0 (0.00)	2 (2.63)
Benazepril group	79	0 (0.00)	1 (1.27)	0 (0.00)	1 (1.27)	2 (2.53)	4 (5.06)
χ^2^							0.135
P							0.713^a^

a , Continuity correction.

## DISCUSSION

This study shows that both LP and benazepril therapy regimens are feasible and effective in treating hypertensive patients with IR. They can safely and effectively regulate blood pressure, blood sugar, and IR. Da Silva AA et al.^15^ showed that hypertension often coexists with glucose metabolism disorders and dyslipidemia, and their common connection is the presence of IR. IR can increase the reabsorption of sodium in renal tubules, thus causing and intensifying the retention of water and sodium, enhance sympathetic nervous activity and blood catecholamine content, and strengthen the action of the renin angiotensin system. Downregulation of Na^+^- K^+^- ATPase activity leads to abnormal membrane ion transport, and a decrease in Ca^+^- ATPase and Na^+^- K^+^- ATPase activities. The increase in calcium and sodium ions in cells can stimulate the transport of sodium and calcium ions, and directly affect vascular smooth muscle cells, causing excessive aggregation of calcium ions in the cells, inhibiting glucose uptake and vasodilation.^16^ Moreover, it can accelerate the growth of vascular smooth muscle, which can increase vascular resistance and blood pressure.^15^,^16^

Zhang et al.^17^ showed that benazepril has positive effects in alleviating IR and lowering blood pressure. Li et al.^18^ also showed that the combination of metformin and benazepril in the treatment of hypertension with metabolic syndrome can help reduce blood lipids, blood pressure, and blood glucose-related indicators, and alleviate IR. Mancusi et al.^19^ indicated that angiotensin II receptor antagonists can block the renin angiotensin system, activate peroxidase proliferators, and activate receptor A. These pharmacological agents, therefore, can regulate glucose metabolism by blocking the renin angiotensin system and activation of angiotensin II receptors.^19^ Nouri-Vaskeh M et al.^20^ found that LP is an angiotensin II receptor antagonist that can selectively act on the AT receptor and inhibit the AT1 receptor subtype of angiotensin II with high selectivity and competitiveness. By selectively blocking the AT1 receptor subtype and directly blocking its effects, LP can effectively inhibit smooth muscle contraction and aldosterone secretion, and has a long-term antihypertensive effect.^20^ Lee et al.^21^ showed that LP can block the binding of angiotensin II to its receptors, thereby blocking the biological effects of angiotensin II, inhibiting inflammatory reactions, promoting microvascular dilation, reopening new blood vessels, and increasing skeletal muscle blood flow and velocity. Furthermore, it increases insulin-mediated glucose uptake and utilization in skeletal muscles, reduces insulin levels, improves insulin sensitivity, and alleviates IR.^21^,^22^ Studies have also reported that losartan treatment is associated with the improvement of insulin sensitivity in hypertensive patients with IR or nondiabetic hypertensive patients.^23^,^24^ These studies have confirmed the application value and related mechanisms of LP and benazepril in hypertension combined with IR, but no comparative studies have been conducted on the differences in their application effects.^15^-^22^ The results of our study confirm that both drugs can achieve good effect when used for hypertension combined with IR. Therefore, in clinical practice, both agents can be selected based on the specific conditions of patients to implement corresponding interventions.

### Limitations:

Firstly, this retrospective analysis is a single center with a small sample size, which may have selection bias. Secondly, no long-term follow-up was conducted, and the research conclusion needs further verification by prospective study with a larger sample size.

## CONCLUSION

Both LP and benazepril have achieved good results in the treatment of hypertension with IR. They can effectively downregulate blood glucose and insulin levels, alleviating IR, and lowering blood pressure of hypertensive patients.

### Authors’ contributions:

**TM:** Conceived and designed the study.

**TM**, **YW**, **HL**, **TW**, **YZ** and **YL:** Collected the data and performed the analysis.

**TM:** Was involved in the writing of the manuscript and is responsible for the integrity of the study.

All authors have read and approved the final manuscript.
